# Supplementary figures and images for: Posteromedial thalamic nucleus activity significantly contributes to perceptual discrimination
Source: PLoS Biol. 2022 Nov 28;20(11):e3001896. doi: 10.1371/journal.pbio.3001896 (PMC9731480; doi:10.1371/journal.pbio.3001896)

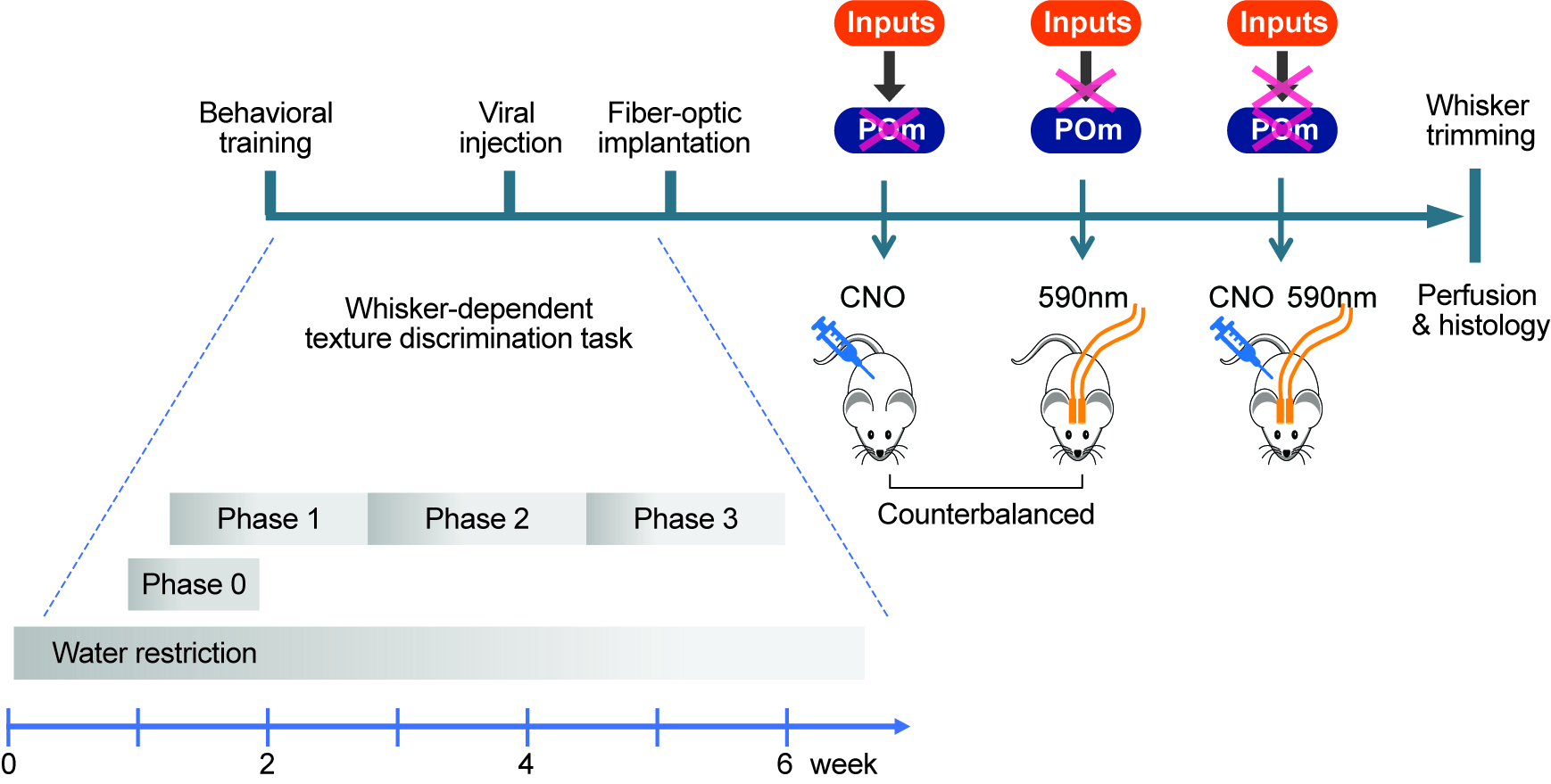

Supplement: S1 Fig — Schematic shows the experimental design, including behavioral task training, surgical procedure, behavioral task with optogenetic or chemogenetic manipulation, whisker trimming, perfusion, and histological evaluation. The different phases of task training are described in the Methods. (TIF) [file pbio.3001896.s001.tif]

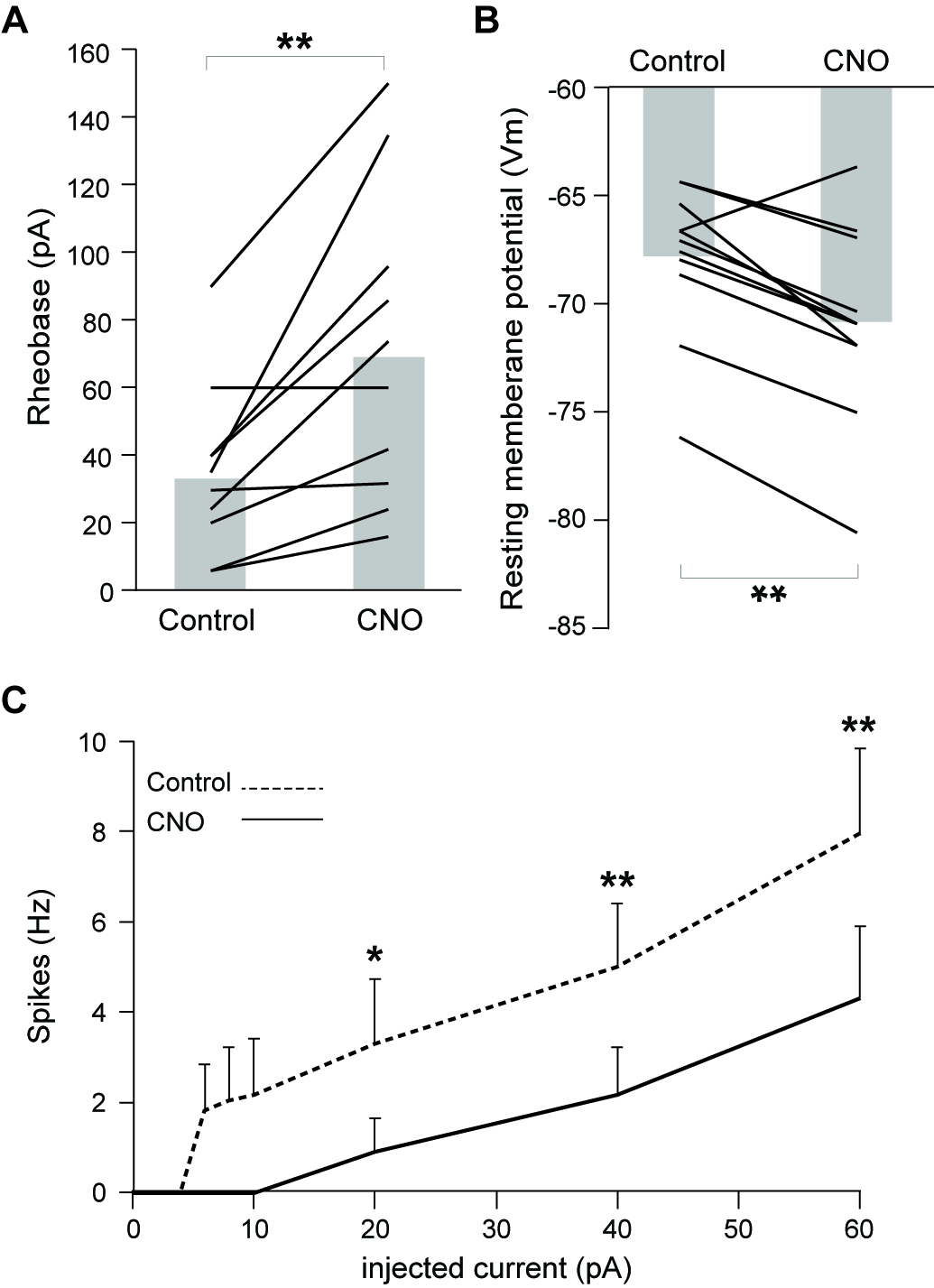

Supplement: S2 Fig — (A) Rheobases of hM4D(Gi)-expressing POm neurons are significantly increased upon CNO application (P = 0.002). Whole-cell patch clamp recording ex vivo was performed on hM4D(Gi)-expressing POm neurons. Gray bars indicate averaged Rheobase across POm neurons, and black lines indicate an individual POm neuron. (B) Resting membrane potentials of hM4D(Gi)-expressing POm neurons are significantly decreased upon CNO application (P = 0.0059). Plotting conventions are same as (A). (C) CNO application decreases evoked spiking activity from hM4D(Gi)-expressing POm neurons in response to current injection. The data in S2A-S2C Fig can be found in S1 Data. (TIF) [file pbio.3001896.s002.tif]

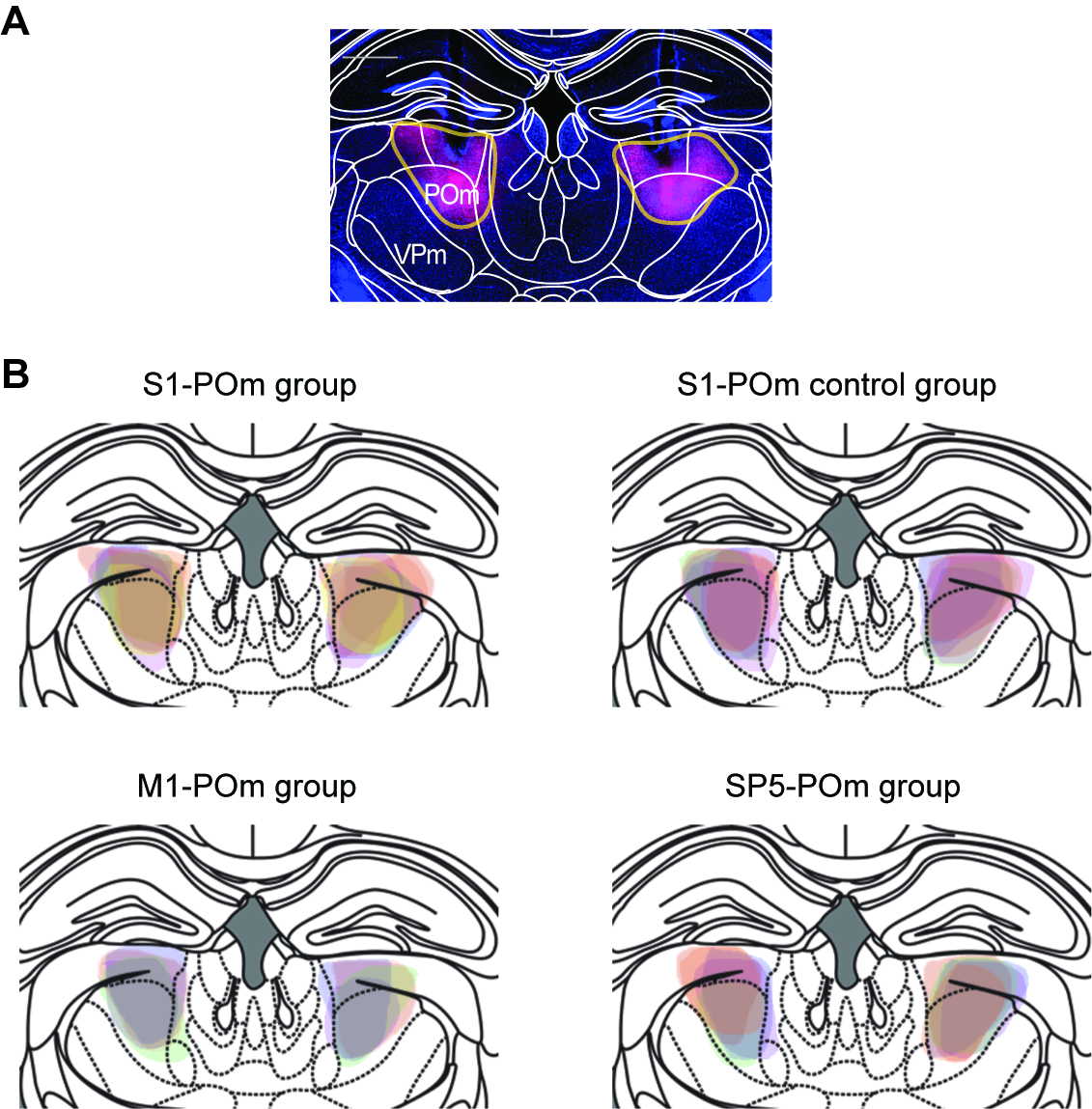

Supplement: S3 Fig — (A) A representative histological image showing the hM4D(Gi)-mCherry expression in POm. The orange line delineates mCherry expressing area. Scale bar = 500 μm. (B) hM4D(Gi)-mCherry expressing areas are overlayed for each group. Each different color of shading represents an individual mouse. (TIF) [file pbio.3001896.s003.tif]

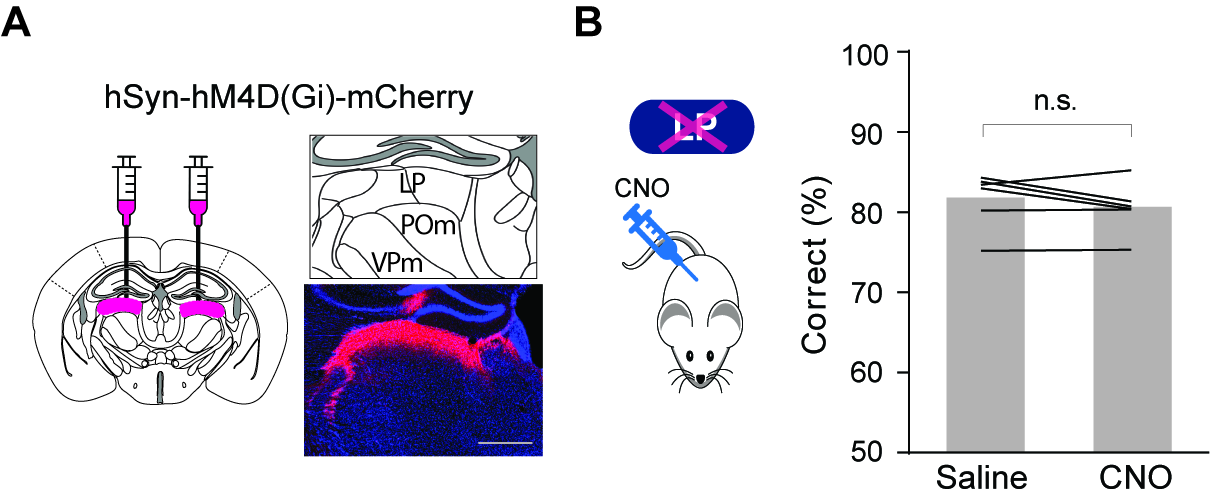

Supplement: S4 Fig — (A) Schematic of experimental design and a representative image showing the expression of hM4D(Gi)-mCherry in LP. Scale bar = 500 μm. (B) Chemogenetic suppression of LP does not affect task performance (P = 0.2434, n = 6, paired t test). The data in S4B Fig can be found in S1 Data. (TIF) [file pbio.3001896.s004.tif]

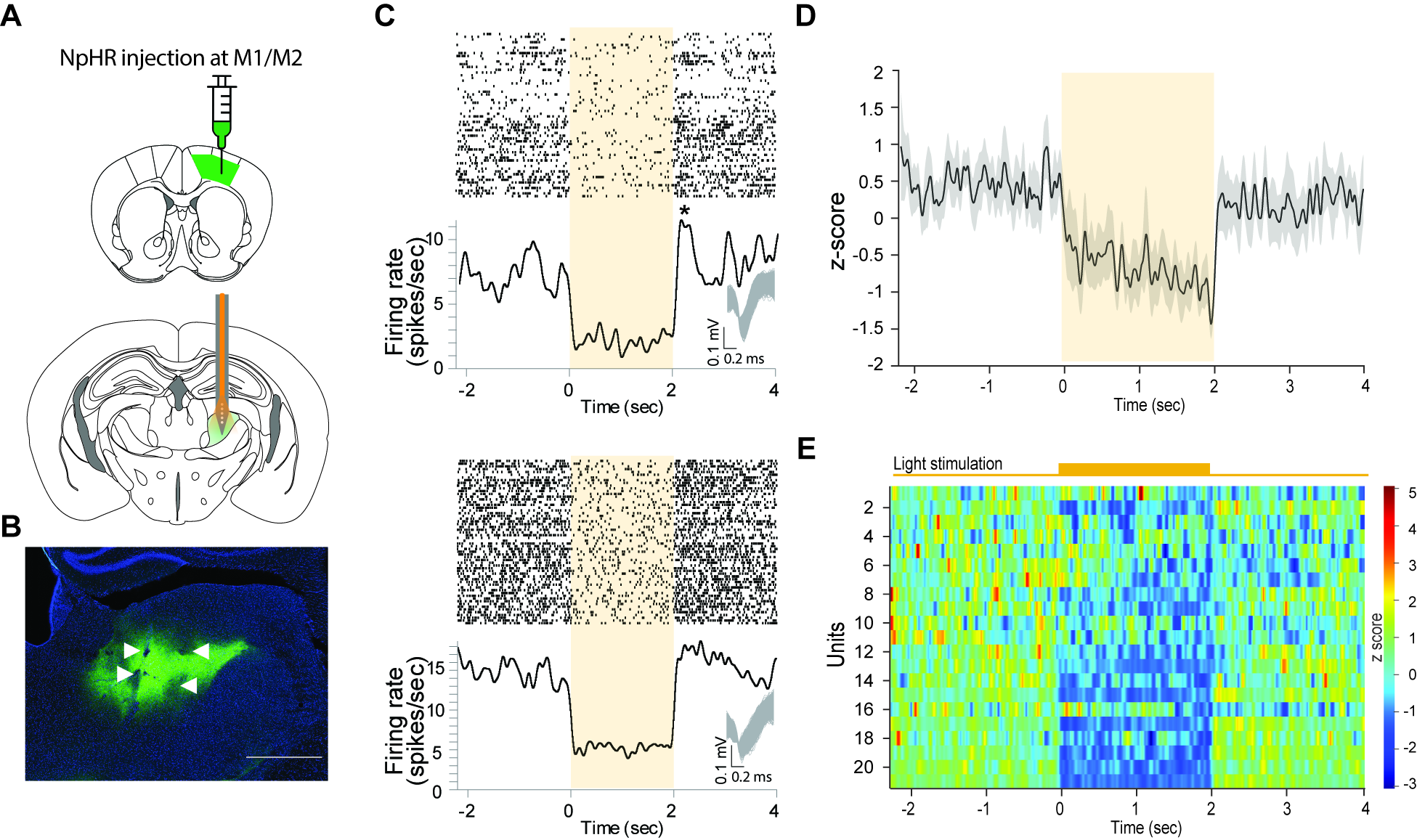

Supplement: S5 Fig — (A) Schematic of optogenetic stimulation and extracellular in vivo recording in POm from awake, head-fixed mice. (B) Representative image showing a recording site. Arrowhead indicates an optrode track. Scale bar = 500 μm. (C) Raster plots and peristimulus time histogram (PSTH) of two example units recorded from POm. 10 ms bin, 60 trials. Shaded areas indicate light stimulation (590 nm, 2 s, 4mW). Illumination with 590 nm significantly decreases the spontaneous spiking activity. The inset shows overlayed spiking waveforms. Asterisk indicates rebound activity upon the termination of light stimulation. (D) Mean spontaneous activity (z-scored) profile of POm units in response to optogenetic stimulation of M1/M2 terminals in POm (21 units). Optogenetic application significantly suppressed spontaneous spiking activity of 21 out of 109 POm units (4 recording sessions from 3 mice). Black line indicates mean, and gray area indicates 95% confidence interval. (E) Heatmap of spontaneous activity of individual POm units. Top orange line indicates optogenetic stimulation. The data in S5C-S5E Fig can be found in S1 Data. (TIF) [file pbio.3001896.s005.tif]

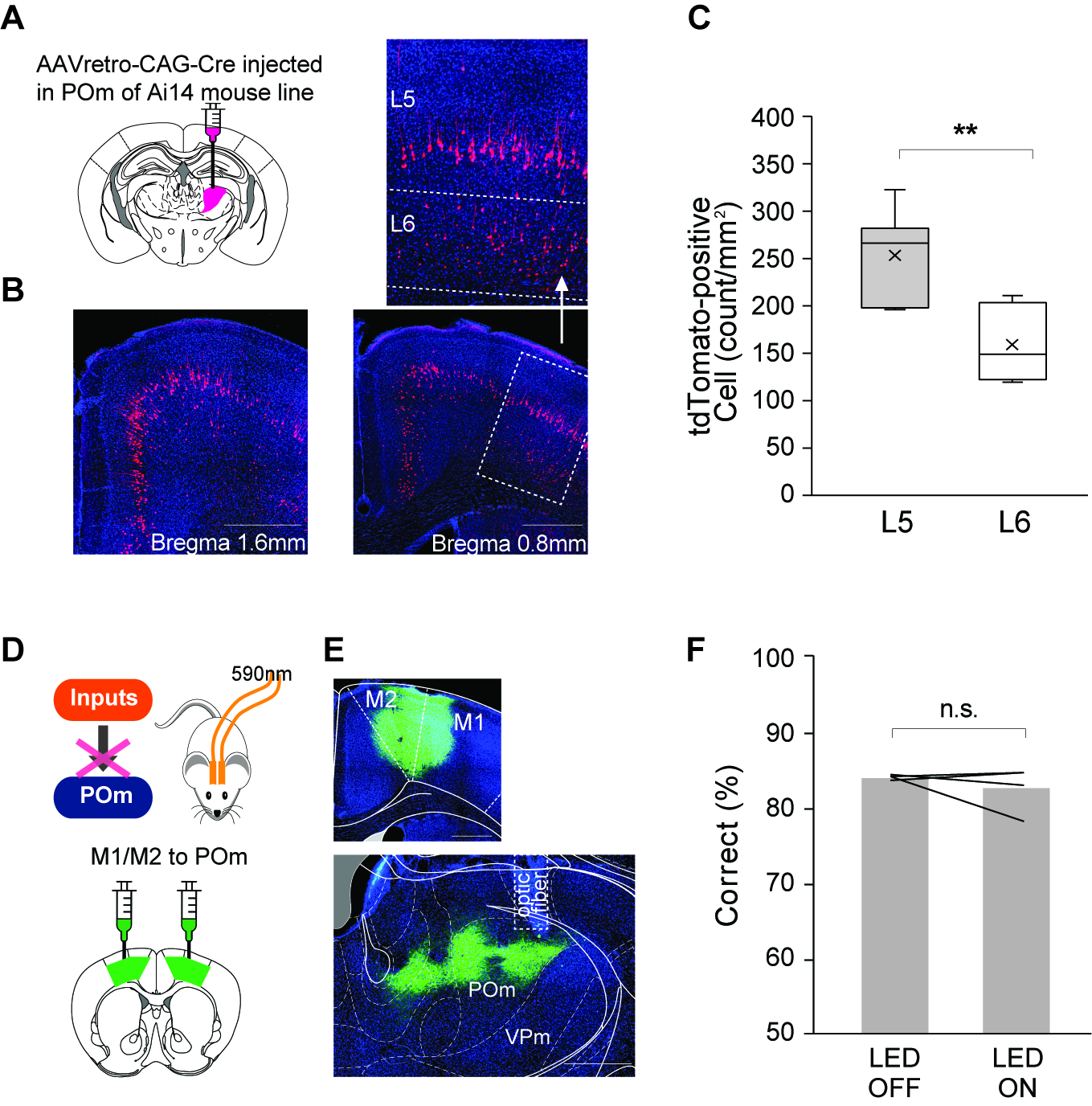

Supplement: S6 Fig — (A) Schematic of experimental design. AAVretro-CAG-cre was injected to POm of Ai14 mouse line to label the neurons projecting to POm. (B) Representative images showing tdTomato-positive neurons in M1/M2. Top image represents a close-up of the L5/6 of M1/M2. (C) The laminar distribution of tdTomato-positive cells within M1/M2 (L5: 251 cells/mm2, L6: 157 cells/mm2, P <0.005, brain tissues n = 6, n = 1 mouse). (D) Schematic of experimental design. Constitutive AAV-hSyn-eNpHR-eYFP was injected to wild-type mice to unbiasedly target L5/6 cells in M1/M2. (E) Representative images showing the expression of eNpHR3.0-eYFP in M1/M2 (top) and the M1/M2 axon terminals innervating POm (bottom). (F) Optogenetic suppression of M1/M2 axon terminals in POm does not affect task performance (P = 0.43, n = 4, paired t test). Scale bar = 500 μm. The data in S6 Fig can be found in S1 Data. (TIF) [file pbio.3001896.s006.tif]

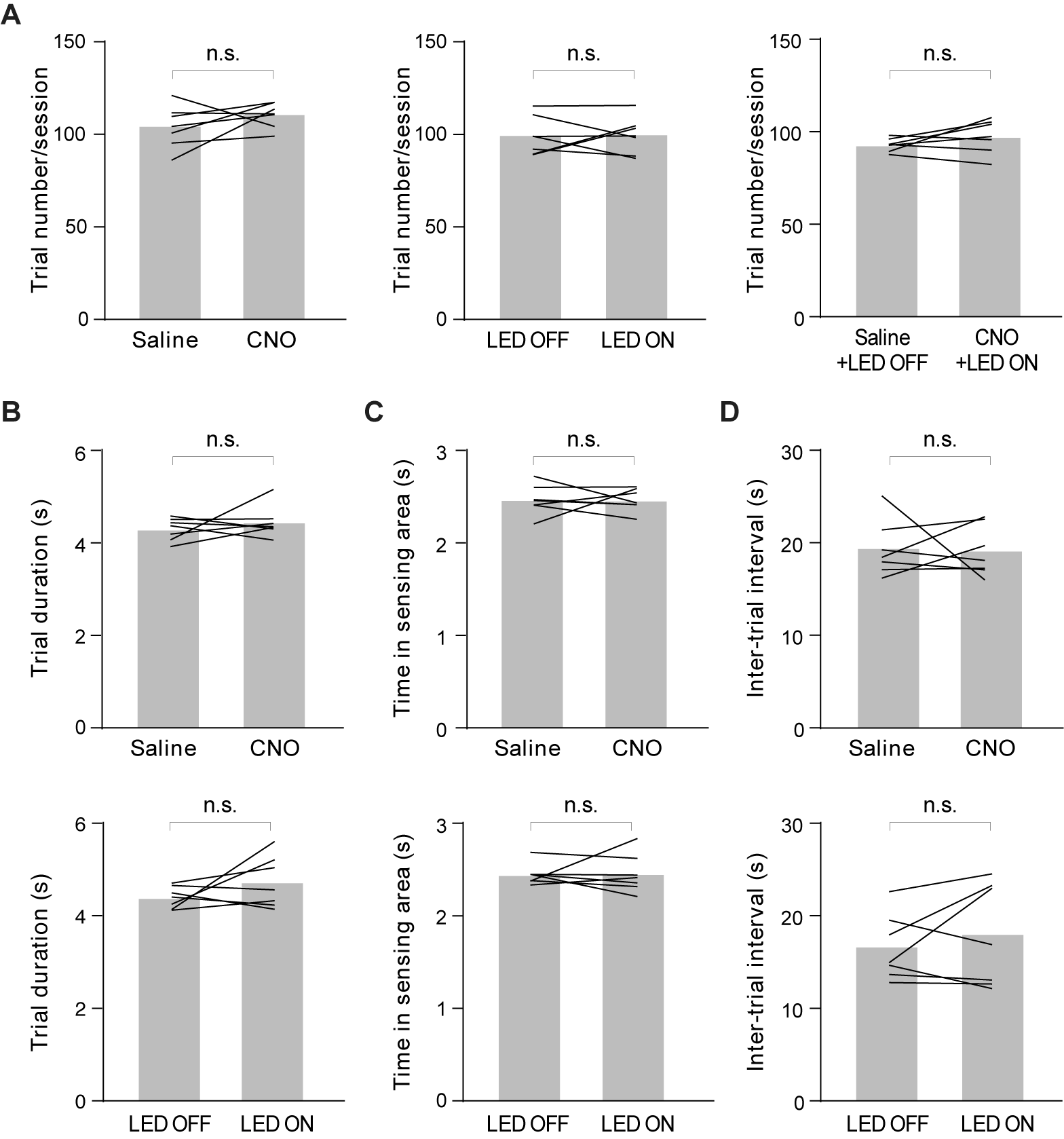

Supplement: S7 Fig — (A) Mice perform a similar number of trials per session under chemogenetic suppression of POm (left), optogenetic suppression of S1 axon terminals in POm (middle), and combined suppression (right). Gray bars indicate average across mice, and black lines indicate an individual mouse. (B-D) Chemogenetic suppression of POm (upper) and optogenetic suppression of S1 axon terminals in POm (lower) do not affect trial duration (B), time spent in sensory zone (C), and the intertrial interval (D). Plotting conventions are same as (A). The data in S7 Fig can be found in S1 Data. (TIF) [file pbio.3001896.s007.tif]
