# Supplementary material for: Posteromedial thalamic nucleus activity significantly contributes to perceptual discrimination
Source: PLoS Biol. 2022 Nov 28;20(11):e3001896. doi: 10.1371/journal.pbio.3001896 (PMC9731480; doi:10.1371/journal.pbio.3001896)
Supplement: Attachment — Submitted filename: JQ Rebuttal Final.pdf [file pbio.3001896.s009.pdf]

RESPONSE TO REVIEWER'S COMMON CONCERNS

We thank the three reviewers for the constructive comments on our manuscript. We have now thoroughly revised the manuscript based on the Reviewers' feedback.

In brief, we performed new lines of experiments to address the following main points in the revised manuscript:

1) We performed positive control experiments to examine the effect of the photo-stimulation of the M1/M2 axon terminals in POm. We demonstrate optogenetic manipulation of the M1/M2 inputs in POm significantly reduced the spontaneous single unit activity recorded from POm (**S5 Fig**).

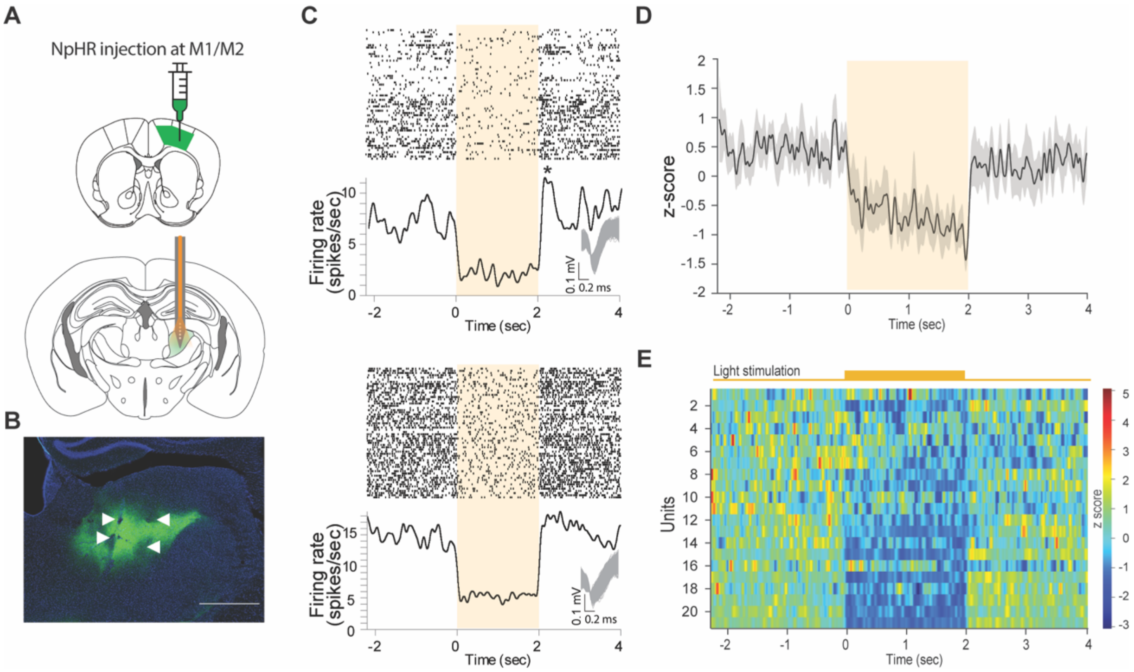

S5 Fig. Optogenetic suppression of M1/M2 axon terminals in POm. (**A**) Schematic of optogenetic stimulation and extracellular in vivo recording in POm from awake, head-fixed mice. (**B**) Representative image showing a recording site. Arrowhead indicates an optrode track. Scale bar = 500 μm. (**C**) Raster plots and peristimulus time histogram (PSTH) of two example units recorded from POm. 10 ms bin, 60 trials. Shaded areas indicate light stimulation (590 nm, 2 sec, 4mW). Illumination with 590 nm significantly decreases the spontaneous spiking activity. The inset shows overlayed spiking waveforms. Asterisk indicates rebound activity upon the termination of light stimulation. (**D**) Mean spontaneous activity (z-scored) profile of POm units in response to optogenetic stimulation of M1/M2 terminals in POm (21 units). Optogenetic application significantly suppressed spontaneous spiking activity of 21 out of 109 POm units (4 recording sessions from 3 mice). Black line indicates mean and gray area indicates 95% confidence interval. (**E**) Heatmap of spontaneous activity of individual POm units. Top orange line indicates optogenetic stimulation.

2) We performed experiments to quantify the laminar distribution of M1/M2 cells that project to POm. While we detected POm projecting neurons from both layers, we found the L5 cells are more abundant than the L6 cells (**S6 Fig**).

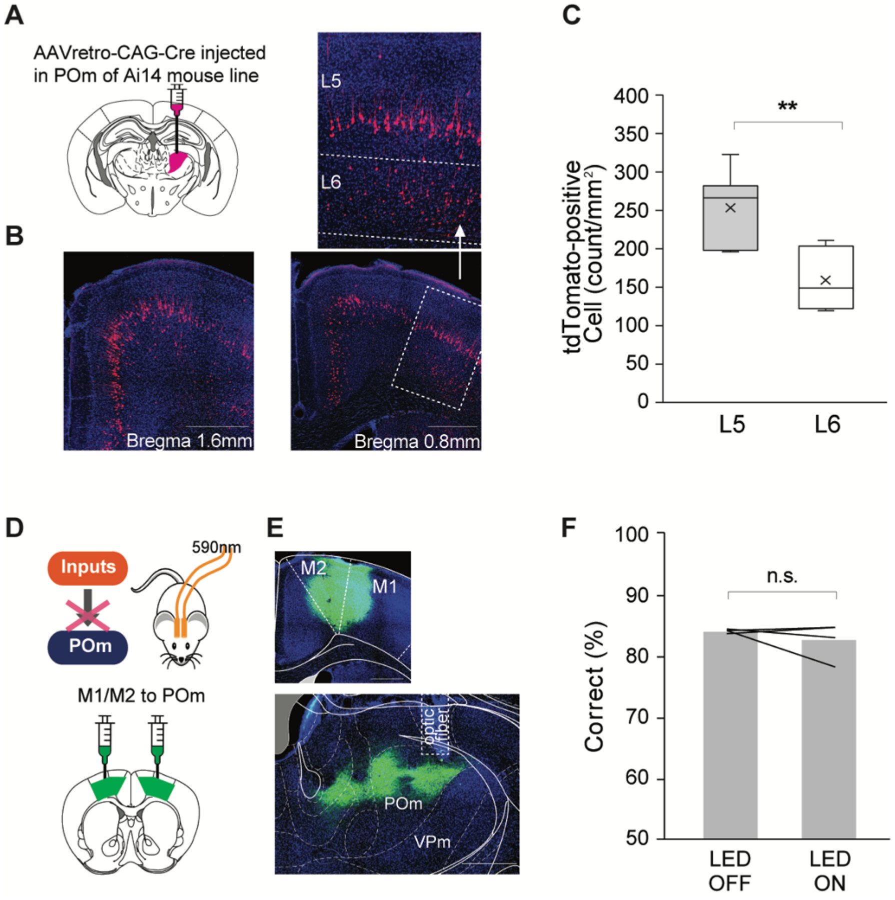

S6 Fig. Projections from M1/M2 to POm. (**A**) Schematic of experimental design. AAVretro-CAG-cre was injected to POm of Ai14 mouse line to label the neurons projecting to POm. (**B**) Representative images showing tdTomato-positive neurons in M1/M2. Top image represents a close-up of the L5/6 of M1/M2. (**C**) The laminar distribution of tdTomato-positive cells within M1/M2 (L5: 251 cells/mm^2, L6: 157 cells/mm^2, P <0.005, brain tissues n= 6, n=1 mouse). (**D**) Schematic of experimental design. Constitutive AAV-hSyn-eNpHR- eYFP was injected to wild-type mice to unbiasedly target L5/6 cells in M1/M2. (**E**) Representative images showing the expression of eNpHR3.0-eYFP in M1/M2 (top) and the M1/M2 axon terminals innervating POm (bottom). (**F**) Optogenetic suppression of M1/M2 axon terminals in POm does not affect task performance (*P* = 0.43, n = 4, paired t-test). Scale bar = 500 μm.

3) We performed experiments to test whether our finding, that M1/M2 inputs to POm do not contribute to the role of POm in sensory perception, is simply because we only targeted L5 Rbp4 cells in M1/M2. We used wild-type mice with a constitutive NpHR to unbiasedly and broadly target M1/M2 cells. Optogenetic suppression of these inputs to POm still did not affect task performance, demonstrating the contribution of M1/M2 inputs to the role of POm in sensory perception is insignificant (**S6 Fig**).

4) We performed positive control experiments to test whether chemogenetic suppression of the thalamic subregion is incomplete, which would imply that the effect of CNO injection in the task performance is insufficient. We targeted VPm with Gi-DREADD (hM4Di) and tested these mice in the sensory perception task. The chemogenetic suppression of

VPm resulted in chance-level task performance, demonstrating our chemogentic application to the subthalamic regions is on target (**updated in Figure 2**).

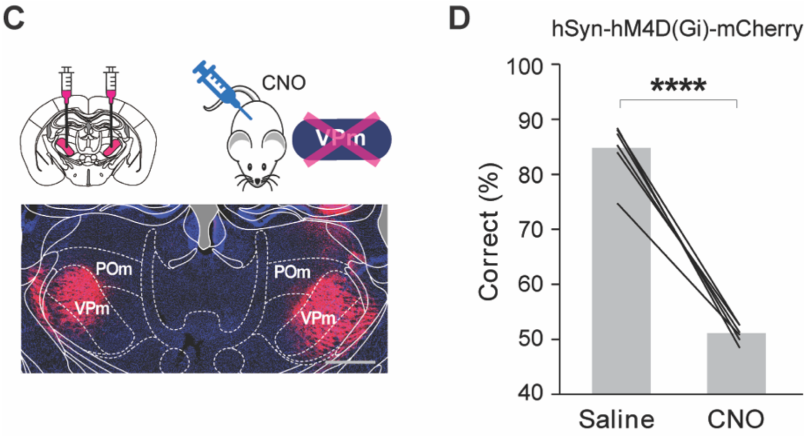

Fig 2. (**C**) Schematic of experimental design and a representative image showing the expression of hM4D(Gi)-mCherry in VPm. (**D**) Chemogenetic suppression of VPm results in chance level performance. ($P < 0.0001$, n = 5, paired t-test). Scale bar = 500 μm.

In addition to these four major revisions, we have addressed all the comments from the reviewers. Please see below for point-by-point responses (reviewer's comment is in blue, our response is in black). We revised the manuscript, after integrating Reviewers' comments and suggestions. Below are the detailed point-by-point responses to Reviewers' comments. We hope the revised manuscript is now acceptable for publication.

REVIEWS:

Reviewer #1: The paper by Qi et al is timely and important. The authors examined the relevance of the posteromedial (POm) nucleus for tactile perception in mice. Indeed, the roles of POm in tactile perception are not yet clear, and are subject to a significant debate in the field. On the one hand, POm neurons receive massive projections from the whiskers (via brainstem nuclei), from S1, and from M1 (directly as well as via a tunable disinhibition circuit of the ZI). On the other hand, their activity depends strongly on the state of the animal (e.g., anesthetized versus awake head-fixed). Thus, the current study is indeed required.
The strategy of Qi et al. for studying the relevance of POm to tactile perception was to train freely moving mice on a 2AFC texture discrimination task and to see how suppressing POm activity affects performance. After showing that the performance of the mice strongly depends on their whiskers, they showed that it also depends on unperturbed operation of POm neurons. Bilateral suppression of POm significantly impaired performance. This study shows that the default execution of the task involves the POm, as bilateral POm activity suppression was induced only during task execution, and suppression sessions were interleaved with control sessions. The anatomical spread of the manipulation was controlled using labeling in each mouse. While this is probably not the best way to control for the suppression of neurons that are

close to the POm-VPM border, it seems good enough - even if only partial suppression of the POm was achieved here, the conclusion still holds. Importantly, in this respect, the authors indeed showed that VPM neurons were not infected (lines 133-136). Then the authors turned to test the dependency of task performance on 3 major inputs to the POm - S1, M1 and Sp5i. The authors conclude that only the S1->POm projection plays a role in this task, because suppressing the other 2 inputs did not affect task performance. Here I find several significant flaws in the design of the experiment, which call for modifying the conclusive statements accordingly. Otherwise, the authors must verify their conclusions with appropriate controls.

We thank Reviewer #1 for the constructive comments. We are pleased that Reviewer #1 agrees the topic is important and timely. To address Reviewer #1's concerns, we have carried out new experiments to bolster and substantiate our conclusions. Please see below.

Major comments

1. Suppression of input from Sp5i. The conclusion of the authors, as stated, requires convincing evidence that this pathway was indeed sufficiently suppressed during the experiment. However, there are significant doubts that this was the case, mostly because: (1) the relevant (rostral) sector of Sp5i (see, for example, El-Boustani et al, 2020; Ebert et al, 2021) might not be sufficiently infected by the virus; (2) the Sp5i terminals in POm may not be sufficiently affected by the laser illumination, which was delivered from fibers located dorsal to the POm. Thus, control experiments, in which the efficiency of suppressing Sp5i terminals is demonstrated, are required here.

To clearly demonstrate our injection site of Sp5i, here we provide a couple example images of PrV, Sp5i and POm. In addition, we also provided our rejection criteria as an example. We hope these additional images address Reviewer #1's concern. As shown in case 1 and 2, the main injection sites of Sp5i are -6.5 ~ -6.8 from bregma. Our injection also did not spread to PrV. In successfully targeted Sp5i brains, we detected Sp5i axon terminals in both VPm and POm as previously reported.

Veinante P, Jacquin MF, Deschenes M. Thalamic projections from the whisker-sensitive regions of the spinal trigeminal complex in the rat. The Journal of comparative neurology. 2000;420(2):233-43.

Chiaia NL, Rhoades RW, Bennett-Clarke CA, Fish SE, Killackey HP. Thalamic processing of vibrissal information in the rat. I. Afferent input to the medial ventral posterior and posterior nuclei. The Journal of comparative neurology. 1991;314(2):201-16.

Williams MN, Zahm DS, Jacquin MF. Differential foci and synaptic organization of the principal and spinal trigeminal projections to the thalamus in the rat. The European journal of neuroscience. 1994;6(3):429-53.

In the failed injection (case 3), injection was too rostral and Sp5i neurons failed to express eYFP, while PrV neurons expressed eYFP. As expected, PrV axon terminals were detected in VPm but not in POm.

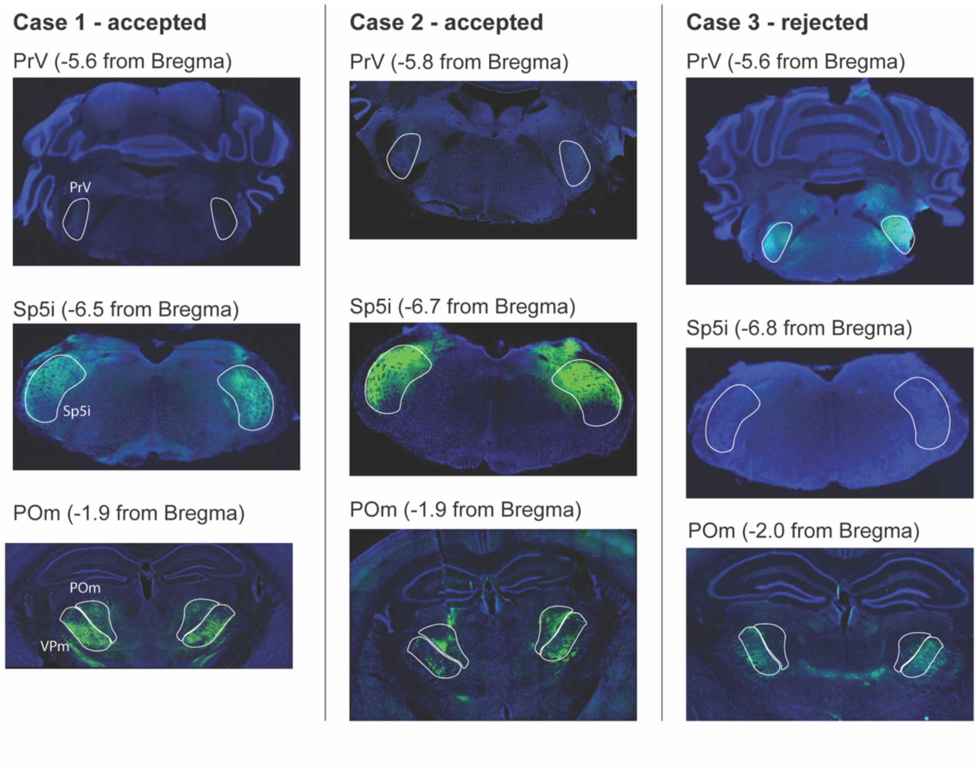

2. Suppression of input from M1/M2. Also here, the conclusion of the authors requires convincing evidence that this pathway was indeed suppressed during the experiment. The major potential problems here are: **(1)** Rbp4-cre may not express Cre in all, or even the most relevant, POm projecting neurons in the motor cortex. Specifically, it is known that corticothalamic neurons in layer L6 of motor cortex project to the POm (Deschenes et al.1998, Harris & Shepherd, 2015, Muñoz-Castañeda et al, 2021); **(2)** the M1/M2, or ZI, terminals in POm may not be sufficiently affected by the laser illumination spreading from the dorsally located fibers. Thus, control experiments, in which the efficiency of suppressing M1/M2/ZI terminals is demonstrated, are required here.

To address Reviewer #1's concerns regarding the role of M1/M2 inputs to POm in sensory perception, we conducted additional experiments.

First, to identify the laminar distribution of M1/M2 neurons that project to POm, we injected AAVretro-Cre (AAV5retro-CAG-Cre) into POm of Ai14 mouse line. With this strategy, the neurons projecting to POm express td-Tomato. We quantified the td-Tomato expressing cells in M1/M2. While tdTomato-positive cells were detected in both layer 5 (L5) and layer 6 (L6) of M1/2, layer 5 cells were significantly more abundant. This result is now reported in **S6 Fig** and lines 239-247.

Second, to further confirm that our main result — that the inputs from M1/2 to POm do not play a role in sensory perception — is not simply due to the partial suppression of M1/M2 inputs to POm, we conducted additional behavior tasks with wild-type mice. We injected constitutive halorhodopsin (NpHR) to L5 and L6 of M1/M2 of wild-type mice to broadly and unbiasedly express NpHR from neurons in L5 and L6 of M1. We carried on the sensory discrimination task with these mice. We report that the optogenetic suppression of axon terminals from both L5 and L6 cells in POm still did not affect the animal's performance in this sensory perception task. These results clearly demonstrate that M1/M2 inputs to POm do not contribute to the role of POm in sensory perception. This result is added to the revised manuscript in **S6 Fig** (please see above) and lines 242-247.

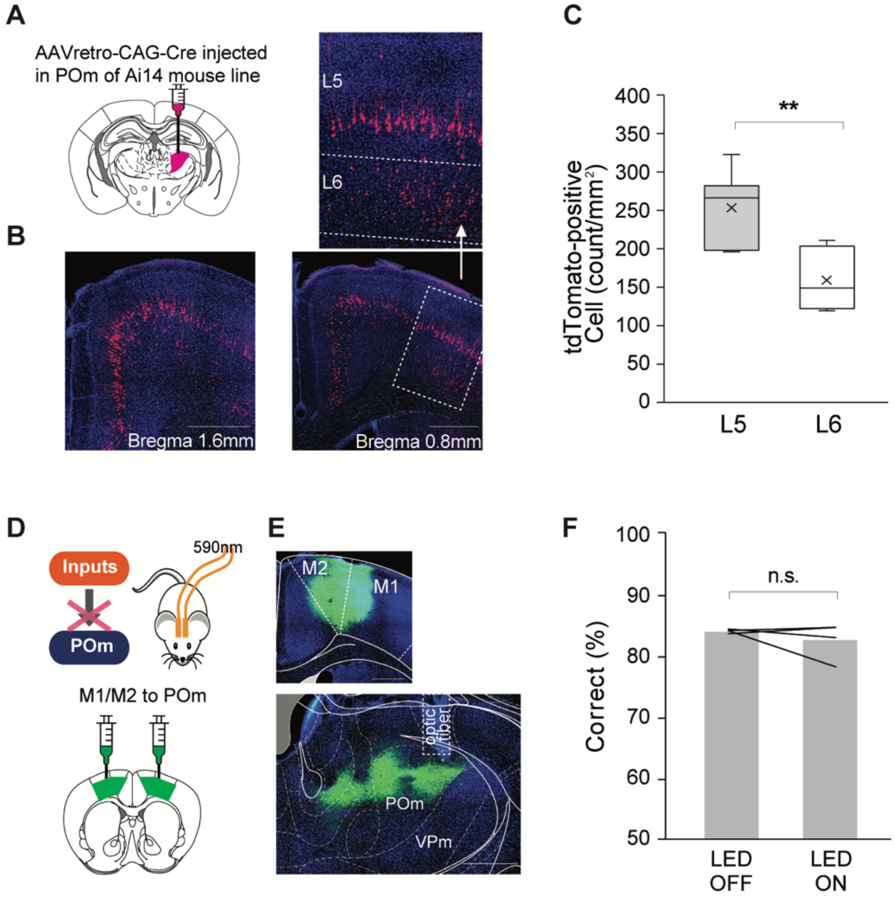

S6 Fig. Projections from M1/M2 to POm. (**A**) Schematic of experimental design. AAVretro-CAG-cre was injected to POm of Ai14 mouse line to label the neurons projecting to POm. (**B**) Representative images showing tdTomato-positive neurons in M1/M2. Top image represents a close-up of the L5/6 of M1/M2. (**C**) The laminar distribution of tdTomato-positive cells within M1/M2 (L5: 251 cells/mm^2, L6: 157 cells/mm^2, P <0.005, brain tissues n= 6, n=1 mouse). (**D**) Schematic of experimental design. Constitutive AAV-hSyn-eNpHR- eYFP was injected to wild-type mice to unbiasedly target L5/6 cells in M1/M2. (**E**) Representative images showing the expression of eNpHR3.0-eYFP in M1/M2 (top) and the M1/M2 axon terminals innervating POm (bottom). (**F**) Optogenetic suppression of M1/M2 axon terminals in POm does not affect task performance (*P* = 0.43, n = 4, paired t-test). Scale bar = 500 μm.

Third, we tested whether our optogenetic manipulation of the M1/M2 axon terminals in POm suppress the activity of POm cells. We extracellularly recorded single unit activity from POm neurons using an optrode under a head-fixed, awake condition. We found that the optogenetic stimulation significantly suppressed the spontaneous firing rates recorded from a subset of single units in POm. We believe that the photostimulation-induced suppression in our recording data is underestimating the optogenetic suppression effect because the diameter of optic fiber used in the unit recording was 100um, which is significantly smaller than the one used in the behavior task (which was 200um). Also, here we performed single unit recording from POm under awake, head-fixed condition, and not during the behavioral task. Therefore, we predict the suppression effect might be more robust during the behavioral task with bilaterally inserted 200um optic fiber. Together, our electrophysiological recording with the optogenetic manipulation demonstrates the efficient suppression of M1/M2 terminals in the POm, thus decreasing POm activity. These data are now added to the manuscript in **S5 Fig** and lines 215-220.

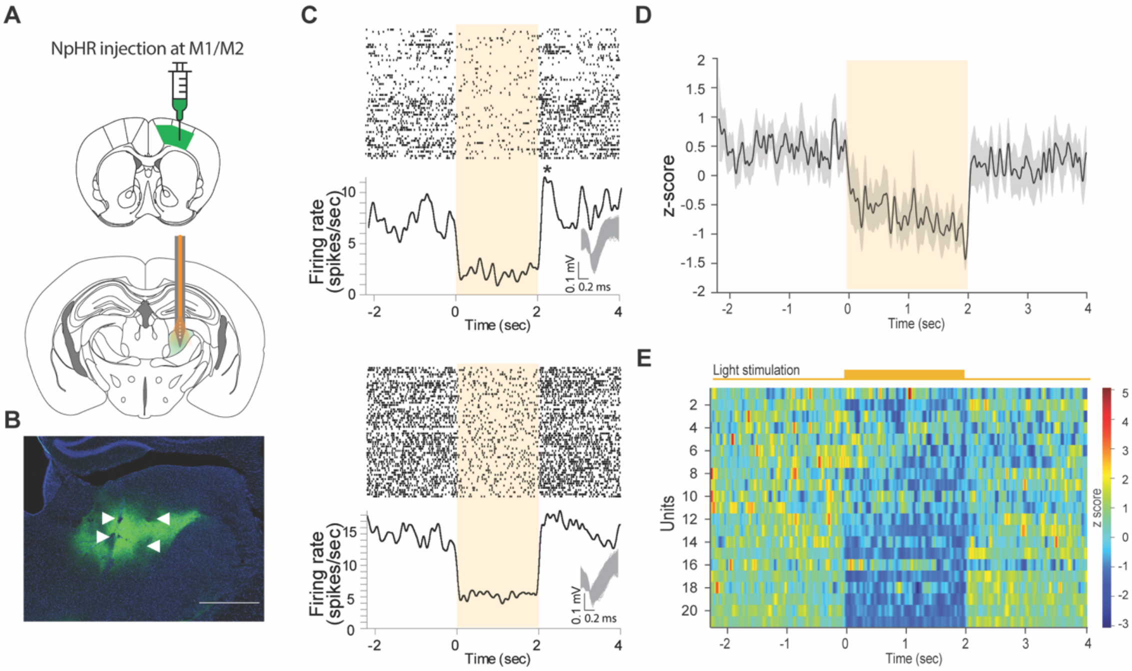

S5 Fig. Optogenetic suppression of M1/M2 axon terminals in POm. (**A**) Schematic of optogenetic stimulation and extracellular in vivo recording in POm from awake, head-fixed mice. (**B**) Representative image showing a recording site. Arrowhead indicates an optrode track. Scale bar = 500 μm. (**C**) Raster plots and peristimulus time histogram (PSTH) of two example units recorded from POm. 10 ms bin, 60 trials. Shaded areas indicate light stimulation (590 nm, 2 sec, 4mW). Illumination with 590 nm significantly decreases the spontaneous spiking activity. The inset shows overlayed spiking waveforms. Asterisk indicates rebound activity upon the termination of light stimulation. (**D**) Mean spontaneous activity (z-scored) profile of POm units in response to optogenetic stimulation of M1/M2 terminals in POm (21 units). Optogenetic application significantly suppressed spontaneous spiking activity of 21 out of 109 POm units (4 recording sessions from 3 mice). Black line indicates mean and gray area indicates 95% confidence interval. (**E**) Heatmap of spontaneous activity of individual POm units. Top orange line indicates optogenetic stimulation.

3. The title should not use the term "higher-order". Rather, simply use "posteromedial" as this is the nucleus that was studied here. There is nothing in the paper that refers generally to "higher-order" nuclei. This distinction is important specifically because there is a debate regarding the "order" of processing among thalamic nuclei - there is substantial empirical evidence that is not consistent with POm being a "higher-order" nucleus (e.g., see Ahissar & Oram 2015). The abstract, and the rest of the paper, can of course refer to the hypothesis that the POm is a "higher-order" nucleus, but as a hypothesis and not as a fact. Referring to it as a fact a) has no empirical justification, b) creates an unjustified bias among the readers, and c) may impair appropriate future empirical designs.

We replaced ‘higher-order’ with ‘posteromedial’ thalamic nucleus in the title, as Reviewer #1 suggested.

Minor comments

1. Lines 48-50. "However, the postulated role of POm in encoding whisking behavior has been challenged with studies demonstrating that POm activity is only weakly modulated by whisker movements [3, 27-30]" - not accurate. The two Yu et al studies cited here (2006, 2015) showed robust responses to whisking, though weaker than those measured in the VPM (see also Groh et al 2013). The authors may want to distinguish here between artificial whisking in anesthetized rats and self whisking in head-fixed animals.

We revised Introduction as Rivewer #1 suggested (lines 46-48).

“However, the postulated role of POm in encoding whisking behavior has been challenged with studies demonstrating that POm activity is weakly modulated by voluntary whisking behavior, although artificial whisking induced by electrical stimulation to the facial nerve of anesthetized animals evokes stronger activity in POm during whisking [3, 27-30].”

2. Fig. 3. Please show the labeling in both hemispheres in the same animals - after all, this is a crucial condition in these experiments.

We replaced the histology figures showing both hemispheres, as Reviewer #1 suggested.

3. A reference to the stereotaxic atlas used in this study should be added.

We added the reference of the stereotaxic atlas (The Mouse Brain in Stereotaxic Coordinates, Franklin & Paxinos, 3rd edition) in Methods (lines 395-396).

4. Specify to which point the DV coordinates refer? Bregma seems to be distanced from Sp5i by more than "3.6-3.9 mm".

We corrected our injection coordinates for SP5i (AP -6.6, ML ± 1.8, DV -3.5, -3.8 mm from pia) (lines 392-393).

Reviewer #2, Laszlo Acsady: The paper by Qi and colleagues addresses an old puzzle in somatosensation i.e. the role of the higher order or paralemniscal thalamic nucleus, (nucleus posteriomedial or Pom) in sensory discrimination. This question has not yet been resolved despite decades of effort put into clarifying the connectivity and synaptic physiology of Pom. Qi et al use a chemogenetic approach to demonstrate that Pom is involved in sensory discrimination during active sensation and utilize optogenetic suppression to identify the role of its excitatory inputs.
The results are overall highly suggestive. Devil is in the details though. While the suppression of correct responses is significant during Pom blockade the performance of the animals is still well above chance level (70%). This can result from:
A) incomplete inhibition of thalamic neurons by CNO,
B) not inhibiting the entire Pom
C) small role of Pom in sensory discrimination.

We thank Reviewer #2 for the positive evaluation and all of the constructive comments.

Version A) can be tested by injecting VPM and test the CNO effect. In this case chance level performance is expected. Or by in vivo recording of unit activity.

To test whether the relatively weak effect of POm chemogenetic suppression in task performance is due to incomplete inhibition of POm cells, we employed the same experimental procedure by injecting hM4D(Gi)-mCherry into VPm and tested the CNO effect on the task performance as Reviewer #2 suggested. Bilateral chemogenetic suppression of VPm with CNO resulted to the chance level performance in the sensory discrimination task. This result suggests that the above chance level performance with POm chemogenetic suppression may not be due to the incomplete inhibition of POm. Since VPm is mostly intact under POm inactivation, it is expected that the animal will perform at above chance levels, as Reviewer #2 pointed out. Our current method does not cover the entire population of POm neurons, thus our manipulation may not fully reveal the role of POm in sensory perception. We now reported the VPm inactivation result in **Figure 2** and Results (lines 130-132) and discussed the potential incomplete suppression of POm activity in Discussion (lines 321-323).

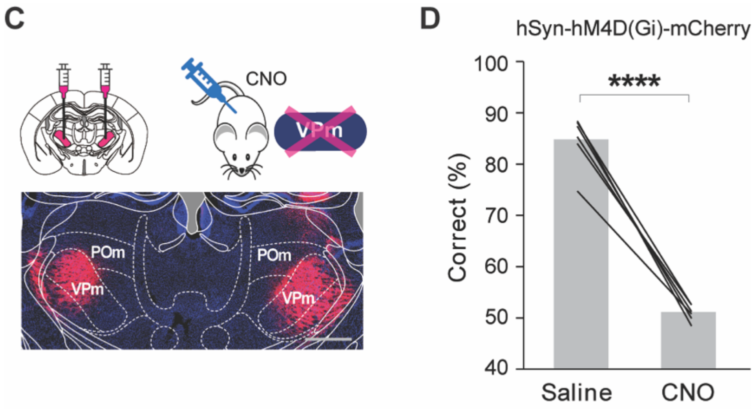

Fig 2. (**C**) Schematic of experimental design and a representative image showing the expression of hM4D(Gi)-mCherry in VPm. (**D**) Chemogenetic suppression of VPm results in chance level performance. (*P* < 0.0001, n = 5, paired t-test). Scale bar = 500 μm.

To exclude B) a much better localization of virus injection site is needed. In order to properly assess the site of CNO action in Pom high resolution, preferably confocal images of the injection sites are needed where it can clearly be determined to what extent Pom was injected. The main problem is that most of the whisker sensitive cells in Pom are located along the VMP/Pom border. This is where S1 layer 5 and Sp5i inputs converge on Pom cells (see e.g Veinante et al. 2000, Lavallee et al., 2005 and Groh et al., 2014 about the convergence). This is even visible on the otherwise very poor quality image of Fig 3E, where the S1 L5 input is clearly more lateral towards the edge of the VPM than the mCherry injection.) The same sector is spared by the injection site on the right side in Fig 2A. this sector is spared by the virus. In addition, we don't know the extent of virus injection at different antero-posterior levels. Concerning option C). If the authors can positively refute A) and B) the authors should appropriately discuss the relatively small effect of Pom and indicate that according to the data sensory discrimination during active sensation may not be the only role of Pom inputs.

We have now presented all the histological images with confocal images that contain both hemispheres, as Reviewer #2 suggested. Our result, that the chemogenetic suppression of VPm *vs*. POm led to a clear difference in the animals' task performance, together with the histological evidence, suggests that our injection into VPm *vs.* POm is mostly on target. While we believe our targeting of two different thalamic regions is specific, we do agree with Reviewer #2's point on the possibility that our manipulation may underestimate the role of POm in sensory perception due to the insufficient inactivation of the entire POm. This potential issue is discussed in Discussion (lines 321-323).

'We limited the viral injection of hM4D(Gi)-mCherry to POm to avoid the contamination of the viral injection to VPm. Therefore, our results may underestimate the contribution of POm in sensory perception.'

Minor points:
It is unclear why "error rate increase" is used in the last chapter (from line 260 on) and not the rate of correct responses. How it is exactly calculated?

Error rate is calculated by subtracting the percentage of correct responses from 100. An increase in error rate is calculated by subtracting the error rate of the correlated control group from the error rate of the manipulation group. We now describe it in the Fig4 legend (lines 298-300)

Optic fiber tracts appear to be artificially made in Fig 3.

We reexamined the optic fiber tracts in Fig 3. We now more accurately marked the optic fiber tracts on the confocal images in Fig3.

Reviewer #3: Qi et al present a punchy account of their investigation into the functional role (if any) of the higher-order somatosensory thalamic nucleus POm in sensory perception. This is an important topic that has been argued over for many years. As the authors correctly note, a probable cause for the lack of resolution has to do with the fact that POm has been studied almost entirely under anesthetized or head-fixed conditions. Here the authors add significant clarity to the field by developing a novel two-alternative forced-choice texture discrimination task for head-fixed mice; this in itself is a noteable contribution to the literature. They then use chemogenetic inhibition of POm to probe for a role in the task and find that performance is reduced. This is an important and, as the authors note, surprising finding. One might think that the animals could perform the task at normal levels entirely with the VPM-based pathway, but this is evidently not so.

Next, the authors used optogenetic inhibition of three different inputs to POm, together with or without chemogenetic inhibition of POm, to assay which if any of these inputs is critical for POm's role in task performance. They find that inputs from S1 L5 are critical in that inhibiting these axons recapitulates and occludes the effect of POm silencing. Testing for occlusion in these experiments is an informative and rigorous aspect of the experiments. In contrast to the effect of S1 axonal inhibition, the authors find no effect of a similar inhibition of the ascending inputs from brainstem to POm, nor of descending inputs from M1/M2 to POm. Finally, the authors show that the deficits associated with POm silencing are more pronounced for more difficult texture discriminations, consistent with a role of POm in processing requiring more attention or vigilance.

We thank Reviewer #3 for the positive evaluation and especially the appreciation of our sensory discrimination task.

Overall, the study seems carefully and rigorously designed and executed. One notable exception to the high overall level of rigor has to do with a lack of positive controls for axonal inhibition. Specifically, given the negative results of the manipulations intended to inhibit axons from SpVi -> POm and from M1/M2 -> POm, how do the authors know that they have in fact obtained inhibition of these axons? It would make a much stronger paper if the authors could somehow provide reassurance on this point. At a minimum, they should acknowledge and discuss it.

The lack of positive controls for axonal inhibition was also raised by Reviewer #1. As described above in response to Reviewer #1, we directly recorded single unit activity from POm neurons using an optrode under a head-fixed, awake condition. We found that optogenetic application of M1/M2 axon terminals at POm significantly reduced the spontaneous firing rates recorded from a subset of single units in POm. We believe that the photostimulation-induced suppression in our recording data is underestimating the optogenetic suppression effect during the sensory discrimination task; 1) the diameter of optic fiber (100 μm) used in the unit recording was significantly smaller than the one (200 μm) used in the behavior task, 2) here we performed single unit recording from POm under a head-fixed awake condition but when the animal was not engaged in the behavioral task. Therefore, we predict the suppression effect may be more robust during the behavior task with 200um optic fiber. Together, our electrophysiological recording with optogenetic manipulation demonstrates the efficient suppression of M1/M2 terminals in the POm, thus decreasing POm activity. These data have now been added to the manuscript in **S5 Fig**.

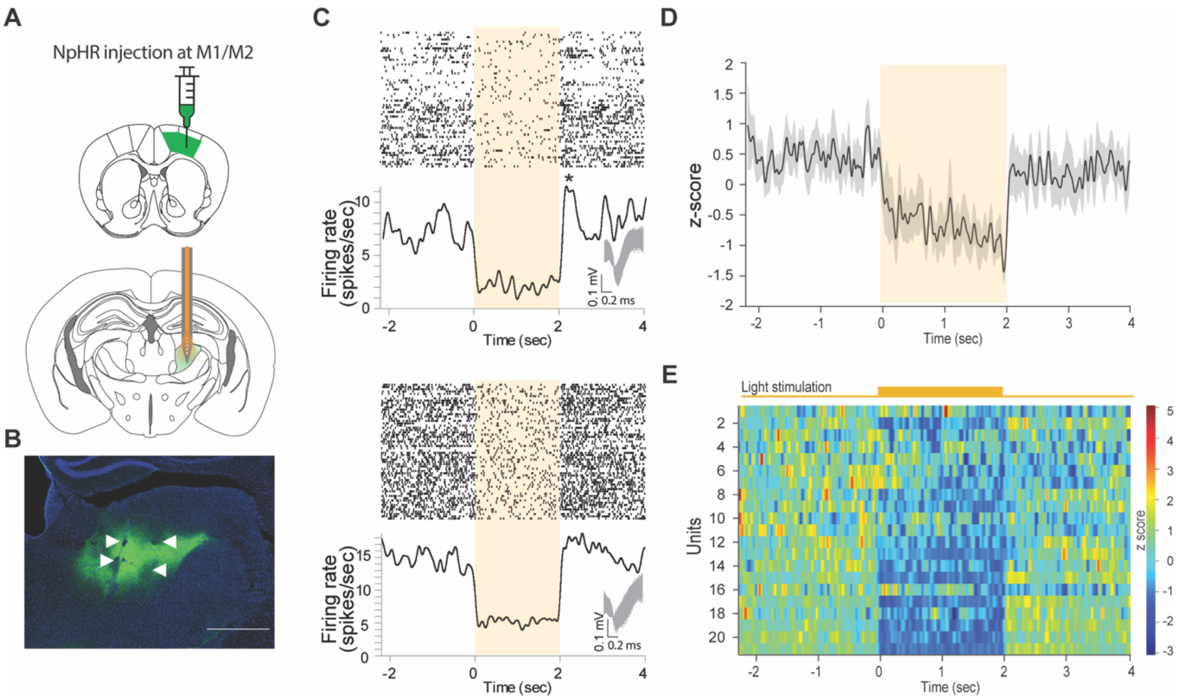

S5 Fig. Optogenetic suppression of M1/M2 axon terminals in POm. (**A**) Schematic of optogenetic stimulation and extracellular in vivo recording in POm from awake, head-fixed mice. (**B**) Representative image showing a recording site. Arrowhead indicates an optrode track. Scale bar = 500 μm. (**C**) Raster plots and peristimulus time histogram (PSTH) of two example units recorded from POm. 10 ms bin, 60 trials. Shaded areas indicate light stimulation (590 nm, 2 sec, 4mW). Illumination with 590 nm significantly decreases the spontaneous spiking activity. The inset shows overlayed spiking waveforms. Asterisk indicates rebound activity upon the termination of light stimulation. (**D**) Mean spontaneous activity (z-scored) profile of POm units in response to optogenetic stimulation of M1/M2 terminals in POm (21 units). Optogenetic application significantly suppressed spontaneous spiking activity of 21 out of 109 POm units (4 recording sessions from 3 mice). Black line indicates mean and gray area indicates 95% confidence interval. (**E**) Heatmap of spontaneous activity of individual POm units. Top orange line indicates optogenetic stimulation.

The authors should also discuss potential caveats associated with their use of halorhodopsin to inhibit axons, which can have unintended effects (B. Mahn, M., Prigge, M., Ron, S., Levy, R., & Yizhar, O. (2016). Biophysical constraints of optogenetic inhibition at presynaptic terminals. Nature neuroscience, 19(4), 554-556. https://doi.org/10.1038/nn.4266).

The Mahn et al. study (2016) raised a concern regarding optogenetic inhibition at presynaptic terminals. In that study, the authors reported that the axon terminals expressing eArch3.0 are paradoxically depolarized upon photo-stimulation. However, the authors did not find significant issues regarding NpHRs expressing axon terminals. In our current study, we used NpHR3.0. The authors reported that both eARch3.0 and NpHRs could evoke rebound activation upon the termination of the photo-stimulation. Our electrophysiological recording of single units from POm clearly demonstrates the suppression of spiking activity upon photo-stimulation *in vivo*. We also detected rebound activity from a few units. We stated the rebound activity in the figure legend (**S5 Fig**).

The writing and overall presentation is quite clear. A couple minor suggestions:
-It would help the reader if the authors showed a schematic of the task sequence in Figure 1A. It is described in the text, but a visual aid would add clarity.

We thank Reviewer #3 for the suggestion. We now revised Fig 1a to clarify the task sequence.

-Section heading titled "The role of POm in sensory perception". I would suggest changing this to "A role of POm in sensory perception". The authors present evidence that POm plays some role, but their data don't have much to say about what exactly this role is.

We revised the section title to "A role of POm in sensory perception", as suggested (line 103).
