# Supplementary material for: Posteromedial thalamic nucleus activity significantly contributes to perceptual discrimination
Source: PLoS Biol. 2022 Nov 28;20(11):e3001896. doi: 10.1371/journal.pbio.3001896 (PMC9731480; doi:10.1371/journal.pbio.3001896)
Supplement: Attachment — Submitted filename: JQ_ MS_Response to Reviewers_10122022.pdf [file pbio.3001896.s010.pdf]

RESPONSE TO REVIEWER'S COMMON CONCERNS

We thank the two reviewers for their positive comments on our manuscript.

Reviewer #1: The authors should be lauded for their extensive efforts to address the reviewers' concerns.
I think that the results are much more convincing now. And thus, it is important to be accurate in drawing conclusions from these results, and specifically conclusions about the possible functions of POm in tactile perception.

As far as my original comments are concerned, we talk basically on the following issue. The authors show that while the integrity of POm is important for performing the task, the integrity of its inputs from M1/M2 and from Sp5i is not. Now let's examine what is the meaning of this result, given existing hypotheses for the functions of the lemniscal and paralemniscal systems.

Regarding the lemniscal system (VPM and S1 in this case) the accepted hypothesis is that it is the major system for processing the spatial features of external objects. This hypothesis is nicely confirmed here.

Regarding the paralemniscal system there are 2 major hypotheses: I. it is the major system for processing/controlling whisker motion during tactile perception, II. It is not conveying any peripheral information that is relevant to tactile perception (and instead the POm is used as a cortico-cortical bridge). Let's see what the current results mean for these 2 hypotheses.

Apparently, the results seem to support hypothesis II, and they are interpreted in this way by the authors. However, careful examination shows that the results cannot discriminate between the two hypotheses.

According to Hypothesis I, lesions of the inputs to the POm should affect the control of whisking, but not texture discrimination. The only way in which such lesions could significantly affect texture discrimination is if they block all relative motion between the whiskers and the object. It could be that performance would also be impaired (slightly, or temporarily) if there is still motion after the lesion but with a modified pattern (modified whisking speed, frequency, amplitude, head-whisker relationships, etc.). Had the authors measured these parameters their comparative analysis could tell us if this was the case and if so, how it affects performance.

Thus, based on their data, the authors are in no position to rule out the possible functions of POm in "sensory perception", as they do. POm can still play a role in sensory perception as suggested by Hypothesis I above. The authors should make this fact clear throughout the article, anywhere where such interpretations are described (I found them in several places). This is crucial in order not to mislead future experimental designs in this field.

We appreciate Reviewer1’s interpretation of our results. While we do agree with the Reviewer regarding the potential roles of POm (hypotheses 1 and 2), we respectively disagree that POm has only one function. Recent studies support the idea that POm may comprise multiple subregions that may each serve different functions. In fact, we described other possible roles for

POm in the previous version of our manuscript, which include sensory-motor integration (Discussion, lines 366-378). In the current version of the manuscript, we specifically restated the potential role of M1 inputs to POm regarding the control of whisking behavior in Discussion (lines 348-353).

Specific points related to same issue:

1. With a 2AFC sensory discrimination task, and performance level ~80%, there are many possibilities for the mice to adapt to motor or motor-related losses. Thus, the mice could easily, and very rapidly, change their motor strategy (all is required is some relative motion between the whiskers and the objects) in order to maintain reasonable performance. Thus, in general in my opinion, a study aimed at addressing vibrissal perception must include careful, high-resolution tracking of whiskers and head kinematics, and their palpation patterns over the textures.

We agree with Reviewer 2 that it would be, in principle, important to analyze whisker and head kinematics under different manipulations. This would be relatively straightforward for head fixed animals with one or a few intact whiskers. We purposely choose instead to use a freely moving condition and keep all whiskers intact to mimic more ecologically natural conditions. Under these conditions, we could not analyze whisker kinematics. In the Discussion section, we now discuss the possibility that animals may rapidly adjust their motor strategy; thus, it may appear that there is no impact of M1 inputs to POm in sensory discrimination, despite the suppression of M1 inputs to POm (lines 348-353).

2. I also think that generalizing from one simple 2AFC sensory discrimination task to "sensory perception" is unacceptable. Statements should refer to the specific task used.

We now use the term 'discrimination' instead of ‘perception’ throughout the manuscript. For example, Lines 58, 60, 140, 157, 188, 192, 196, 214, 264, 283, 341, 346: ‘perception’ to ‘discrimination’. Lines 109, 169-170, 171-172, 180-181, 286, 340: ‘sensory perception’ to ‘perceptual sensory discrimination’. Lines 260-261: ‘sensory perception’ to ‘texture discrimination’.

Reviewer #2: The authors adequately answered all my concerns. Congratulations for the revision. Nice work.

We thank Reviewer 2 for such a highly positive evaluation.
